# Punctate Midline Myelotomy Reduces Pain Responses in a Rat Model of Lumbar Spine Pain: Evidence that the Postsynaptic Dorsal Column Pathway Conveys Pain from the Axial Spine

**DOI:** 10.7759/cureus.2371

**Published:** 2018-03-26

**Authors:** Haring J Nauta, Sabrina L McIlwrath, Karin N Westlund

**Affiliations:** 1 Neurosurgery, University of Louisville; 2 Anesthesiology and Critical Care Medicine, University of New Mexico School of Medicine

**Keywords:** nociception, back pain, hypersensitivity, myelotomy, sensory pathways, spinal cord stimulation, alternatives to chronic opiate therapy, facet joint pain, surgery for pain

## Abstract

Punctate midline myelotomy (PMM) has been successfully applied clinically in humans for the relief of intractable visceral pain. The operation is thought to work by interrupting the postsynaptic dorsal column pathway (PSDC) of the spinal cord. In fact, PMM was developed specifically for that purpose after it was demonstrated in rats that the PSDC conveyed about 90% of the visceral pain information to the thalamus. The application of PMM also to the problem of severe intractable back or spine pain was never tested, and it has never been established whether the PSDC pathway relates only to visceral pain or whether there may be a broader involvement with pain affecting structures of embryological midline origin, perhaps including the spine. Retrospective analyses of decades of results from various attempted myelotomy procedures in man for the relief of pain are consistent with the notion that the common element crucial to the successful midline or visceral pain relief was the interruption--even incomplete--of the PSDC pathway. Herein, we present evidence from a rat model of lumbar facet pain that interruption of the PSDC significantly reduces pain responses. The implications for the possible treatment of severe intractable spine pain in man are discussed.

## Introduction

The purpose of this study is to clarify how pain signals originating in the spine, specifically from lumbar facet joint inflammation, ascend in the spinal cord to reach higher levels of the nervous system. Until the discovery of the postsynaptic dorsal column (PSDC) pathway [1], the answer to this question was presumed to be trivial because it was believed that all pain signals reach the thalamus in some component of the classical spinothalamic tract (STT) system, ascending bilaterally crossed in the anterolateral quadrants of the spinal cord. The dorsal columns were believed to be reserved for the “epicritic” sensations, including vibration, position, and light touch. The major role of the dorsal column midline in visceral pain conduction went unrecognized. More recently, the view that there is only one major pain tract system (however subdivided) has been superseded, and it is now abundantly clear from both clinical and laboratory studies that there is a robust pain pathway that ascends in the dorsal column midline and largely serves visceral pain [2]. Laboratory studies in rats by Al-Chaer et al. [3] and others have defined this PSDC pathway in more detail [4]. Retrospective analyses of clinical patient outcomes following various older myelotomy procedures affecting the dorsal columns were also consistent with this view [2, 5].

As it became understood that the PSDC is the principal pathway for conduction of information about visceral pain, the punctate midline myelotomy (PMM) was developed specifically to interrupt this pathway for the treatment of severe intractable visceral pain [6-7]. There is now growing acceptance that PMM can relieve severe intractable visceral pain refractory to conventional treatment methods with acceptable postoperative sequela and that PMM (or other myelotomy interrupting the PSDC) has at least a niche role in clinical neurosurgery for this purpose [2, 5-10].

The contrast between the visceral pain conducting PSDC pathway and the somatic pain conducting STT pathway has been investigated in multiple studies [2-3, 11-15]. The place of spine pain in this dichotomy has not been evaluated. This is a major gap in our knowledge since spine pain is such a major and common problem clinically worldwide [16]. It remains unclear if pain originating in the spine would follow the pattern of visceral pain or that of somatic pain in terms of the spinal cord pathways followed. Because the spine shares some similarities to the viscera as a deep, midline originating structure, it would not be surprising to find that the spine itself was served by the PSDC in what we now consider a pattern typified by visceral pain. On the other hand, because of its mesenchymal origin, the spine could plausibly follow the pattern more typical of somatic pain, ascending predominantly or even exclusively in the STT system of pathways. Of course, some pattern of distribution to both PSDC and STT systems is also possible. In this study, we present evidence that the PSDC may play a role in conveying the pain of axial spine origin. 

## Materials and methods

Animals

All animal procedures were approved by the University of New Mexico Institutional Animal Care and Use Committee (IACUC Protocol #200613) and were conducted in accordance with the guidelines of the International Association for the Study of Pain for the Ethical Treatment of Experimental Animals [17].

Eleven Sprague Dawley rats (Harlan Laboratories, Indianapolis, IN), age 8 weeks, were housed individually on a 12/12 hour reverse light cycle so that behavioral assays were conducted during their active night phase. Animals were given food and water ad libitum.

Lumbar Facet Joint Osteoarthritis Using Intraarticular Urokinase Plasminogen Activator Injection

The present lumbar facet osteoarthritis procedure was devised after consulting two similar procedures [18-19]. Sprague Dawley rats were anesthetized with isoflurane (5% for induction, 2.3 - 2.5% during injection) and placed in a prone position. The lumbar spine was palpated to identify the L5 and L6 spinous processes. A Hamilton microsyringe (Hamilton Co., Reno, NV) mounted with a Luer lock to a 30 gauge, 1-inch length needle was used for the injection. The needle tip was inserted percutaneously into the posterior medial facet joint articular cavity for injection of 5 µL serine protease urokinase plasminogen activator (uPA) (2 mg/L in 0.9% saline; EMD Millipore-CC4000 (EMD Millipore Sigma, Burlington, MA, USA). The needle was held in place for 15 seconds post-injection before removal to reduce leakage. Intraarticular injections were made bilaterally.  

Surgical Lesion of the Postsynaptic Dorsal Column Pathway

**Figure 1:**
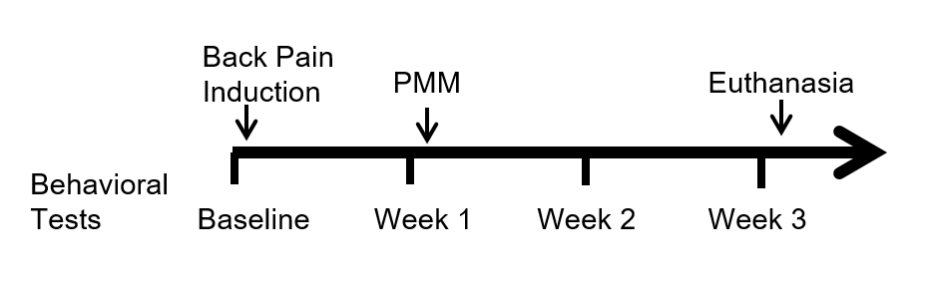
Timeline PMM: punctate midline myelotomy

After one week, rats were re-anesthetized with isoflurane (1-2%)/oxygen (1-2%), and a surgical skin incision and laminectomy were done to expose the thoracic spinal cord (T5-T7). Bilateral needle (30 gauge, 1/2 inch length) punctures in the spinal cord midline dorsal column to a depth of 1 mm were done to disrupt the postsynaptic dorsal column pathway. The skin incisions were closed with a nylon suture followed by application of a triple antibiotic to the site. Figure 1 summarizes the sequence and timing of the procedures and testing.

Assessment of pain-related behaviors

Animals were acclimated to the testing room in their home cage for one hour prior to behavioral tests. Tests for both mechanical threshold and Hargreaves heat sensitivity were performed one week prior to induction of the back pain model (Day 0) and at Days 3, 7, 14, and 21 post-back pain model induction.

Von Frey Mechanical Threshold Assessment

Animals underwent behavioral testing to determine baseline mechanical sensitivity threshold using the up-down method [20]. Animals were placed on an elevated metal mesh (3 mm^2^ holes) in individual clear lucite boxes and the hind paw glabrous skin was probed using a graded series of calibrated von Frey filaments. Withdrawal of the foot from the von Frey filament stimuli is considered a positive response. The responses to the filaments of graded gram force strength were applied to an algorithm determining the mechanical sensitivity threshold for comparisons among the experimental conditions.

Hargreaves Heat Sensitivity Assessment

Thermal sensitivity was measured by the latency of the withdrawal response to radiant warming stimuli in the noxious range as previously described by Hargreaves et al. [21]. Animals were placed in plexiglass boxes on an elevated glass plate under which a radiant heat stimulus was applied as a concentrated beam of light onto the foot pad. Animals were free to shift position to avoid the stimulus at any time. The light beam was automatically turned off when the animal withdrew, allowing the measurement of time between the start of the light beam and the withdrawal event. Five minutes were allowed between each trial and three trials averaged with a one-hour maximum acclimatization and testing time limit in the cubicle. 

Statistical analysis

Data are expressed as the mean ± standard error of the mean (SEM). Statistical significance of behavioral data was determined using Student t-test with the significance level set at *p *< 0.05.

Histology

Animals were deeply anesthetized using pentobarbital (40 mg/kg) and transcardially perfused with 0.9% heparinized saline followed by 4% paraformaldehyde (4% PFA) in phosphate buffered saline (PBS). The thoracic spinal cord was excised, post-fixed, cryoprotected using a 30% sucrose solution, and embedded in optimal cutting temperature (OCT) compound (VWR, Atlanta, GA, USA). The tissue was sectioned into 25 μm sections and collected in PBS. Free-floating sections were reacted overnight with primary antibodies rabbit anti-Iba1 (1:1000; Wako, Richmond, VA, USA) and guinea pig anti-NeuN (1:3000; Cat. # ABN90, EMD Millipore Sigma, Burlington, MA, USA) at room temperature. The tissue was washed and incubated for two hours in secondary antibodies (1:1000 goat anti-rabbit IgG conjugated with Alexa Fluor 488 and 1:1000 goat anti-guinea pig IgG conjugated with Alexa Fluor 647) (Molecular Probes, Eugene, OR, USA). The tissue was mounted on glass slides and coverslipped with Vectashield mounting medium with DAPI counterstain for cell nuclei (Vector Labs, Burlingham, CA, USA). Stained tissue was visualized using an Olympus BX61WI microscope and Olympus FluoView FV1200 confocal microscope system (Olympus America, Melville, NY, USA).  

## Results

Testing was initially done for baseline evoked reflexive mechanical and heat responses standard in pain research that was otherwise not detectable by observation. This included testing with von Frey nylon fibers to determine mechanical withdrawal threshold on the hind paw and the Hargreaves test of heat sensitivity. After induction of lumbar facet joint osteoarthritis (OA) and a brief recovery time, all the animals appeared normal, active, and well-groomed. The lumbar facet joint OA model induces measurable changes in evoked reflexive responses to both mechanical and heat stimuli. Footpad sensitivity is only observed with behavioral testing methods standard in the field, as approved by the American Pain Society and the International Association for the Study of Pain.  

**Figure 2:**
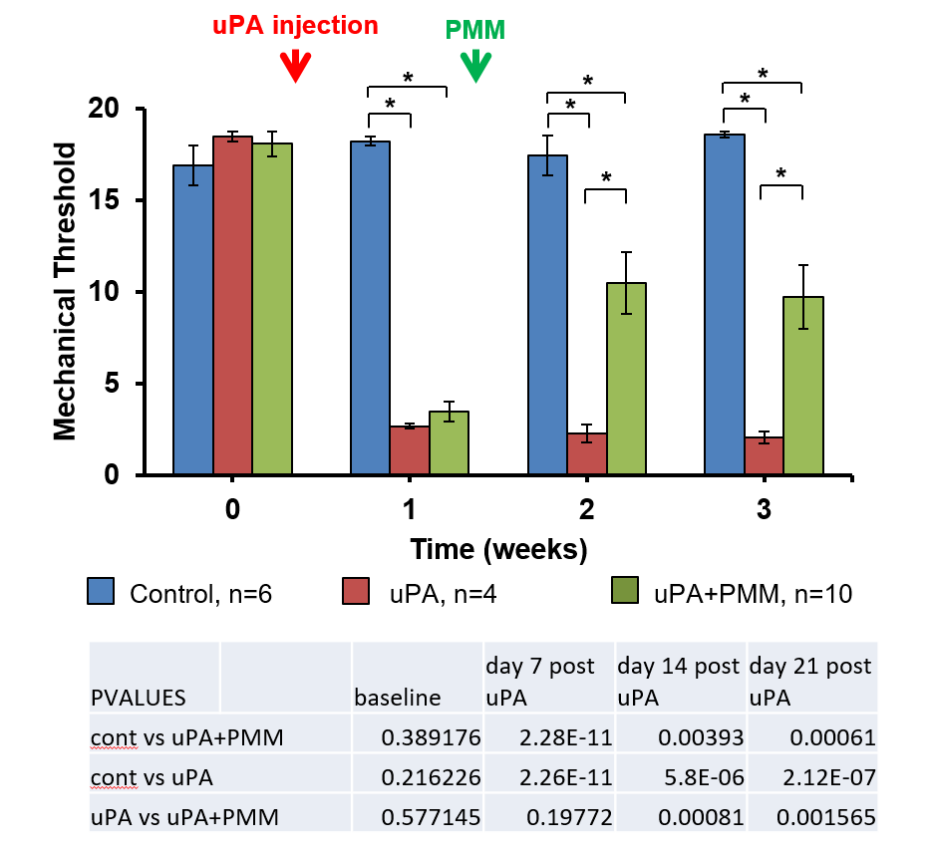
Mechanical Threshold Testing Bilateral injection of uPA into lumbar facet joint produced mechanical hypersensitivity of the hind paws. Mechanical withdrawal thresholds were determined independently on each hind paw in a total of 10 animals. uPA: urokinase plasminogen activator; PMM: punctate midline myelotomy

Mechanical sensitivity testing was performed for comparisons of responses before and after uPA injections made bilaterally into the lumbar facet joints. After induction of OA, the mechanical withdrawal thresholds in the uPA + PMM group were significantly decreased from 18.1 ± 0.7 g at baseline to 3.5 ± 0.6 g on Day 7 (p < 0.001) after uPA injection (Figure 2). Similarly, mechanical withdrawal thresholds in the uPA-only group were significantly decreased from 18.5 ± 0.3 g at baseline to 2.7 ± 0.2 g on Day 7 (p < 0.001). On Day 14, one week after PMM, mechanical withdrawal thresholds significantly increased to 10.5 ± 1.7 g (p < 0.005), while they were unchanged at 2.3 ± 0.5 g in the uPA-only group. Two weeks after PMM, mechanical withdrawal thresholds remained increased at 9.7 ± 1.7 g.

**Figure 3:**
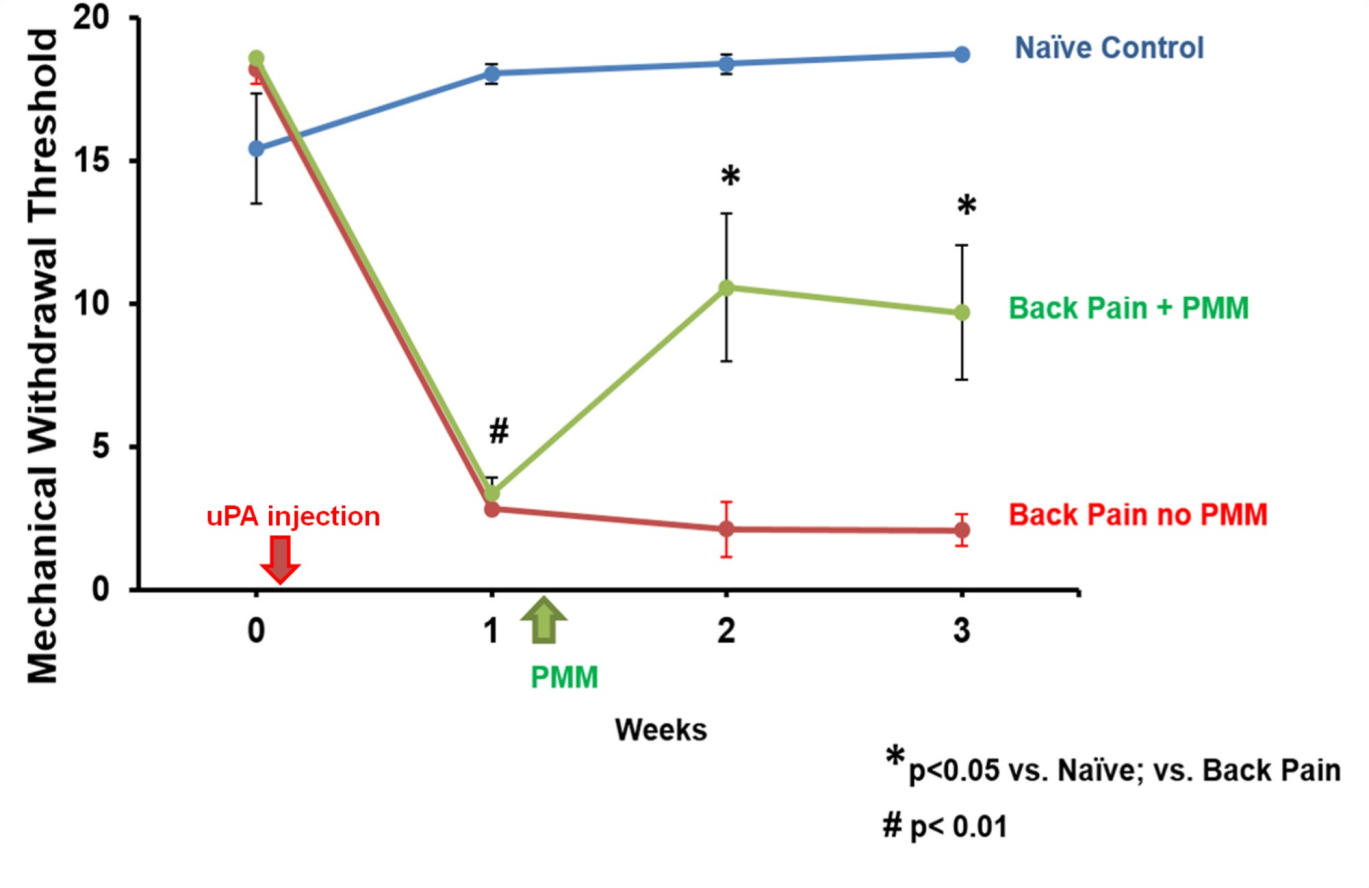
Mechanical Threshold Testing Results Summary The mechanical thresholds are shown here as a timeline. uPA: urokinase plasminogen activator; PMM: punctate midline myelotomy

Heat sensitivity, measured as the response latency to a shift away from the radiant heat source directed onto the hind paw, was reduced from 13.1 ± 1.1 s on the left and 13.6 ± 0.6 s on the right hind paw at baseline to 10.5 ± 1.4 s and 9.3 ± 0.8 s on Day 3 and 10.2 ± 0.6 s and 9.7 ± 0.5 s on Day 7 after OA model induction (Figure 3). After surgical PMM, hypersensitive responses to heat stimuli were significantly attenuated. Heat response latencies returned to baseline after PMM (left: 13.5 ± 0.5 s; right: 13.8 ± 0.8 s).

These data indicate that the hypersensitivity induced by the lumbar facet arthritis was significantly reduced for heat-related, pain-related behavior after PMM. The hypersensitivity in this model untreated reportedly persists for four weeks [19]; thus, the dorsal column spinal cord lesion was effective. This provides evidence that lumbar spine pain signaling was traveling in the dorsal columns.

**Figure 4:**
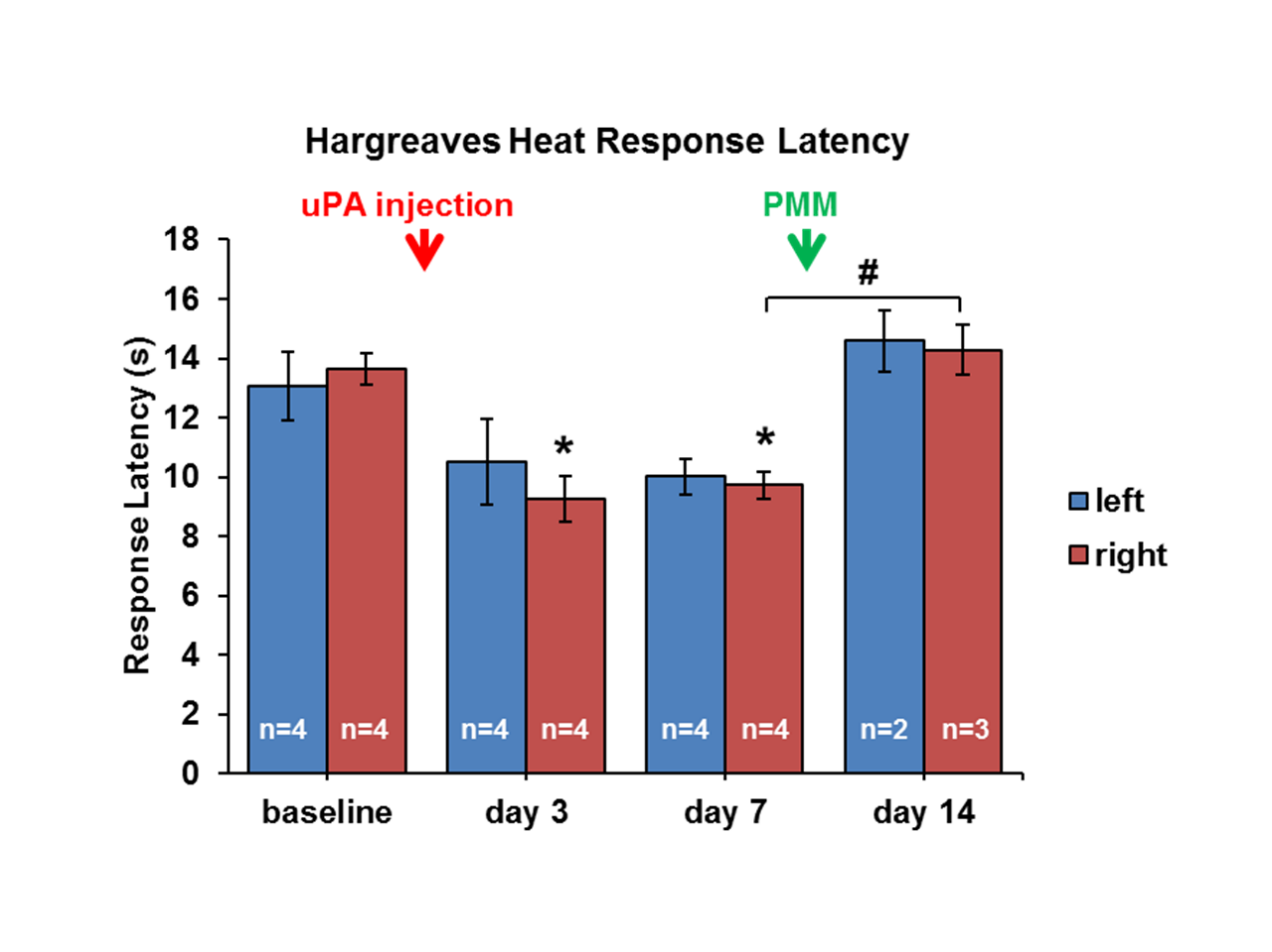
Hargreaves Testing Bilateral injection of uPA into the lumbar facet joint produced heat hypersensitivity of the hind paws. The heat response latency using the Hargreaves test determined hypersensitivity on Days 3 and 7 which was alleviated by the PMM. * p < 0.05 comparison to baseline; # p < 0.05 comparison between Days 7 and 14 n = number of animals per time point; uPA: urokinase plasminogen activator; PMM: punctate midline myelotomy

Histological verification of the PSDC lesion was done at the end of the study. Examples are provided in Figure 4 of spinal cord sections from a rat with a PSDC lesion and one control without a lesion, shown as merged and single views of staining for neurons, microglia, or nuclei counterstained. Both low and high power magnifications are provided.

**Figure 5:**
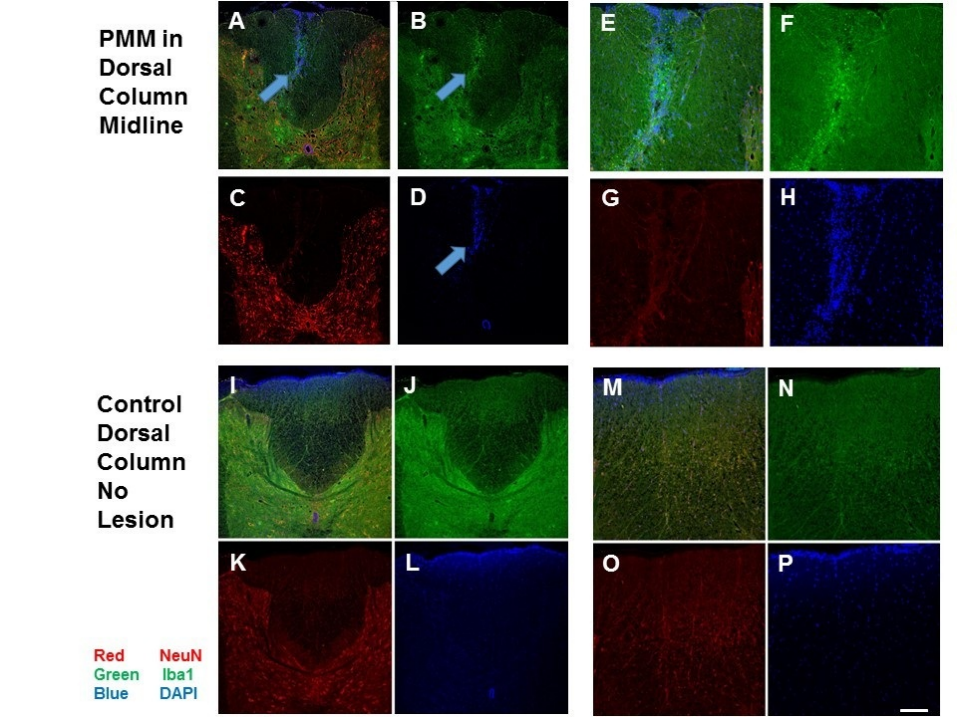
Spinal Cord PMM Lesion Histology with Arrows A-H: Microglia (arrows) are associated with the PMM lesion in the dorsal white matter of the spinal cord midline after two weeks (three weeks after OA induction) shown at low (A-D) and high (E-H) power. I-P: For comparison, the dorsal portion of the spinal cord of a naïve control animal is shown at low (I-L) and high-power (M-P). A, I: Computer-generated images combining dual NeuN (red, C, K), neuronal, and IBA1 (green, B and J) immunofluorescent images counterstained with DAPI (blue, D, L). The spinal cord coronal section was reacted with IBA1 antibody to identify microglia (green). B: Microglia were observed at the lesion site two weeks post-PMM (green). P: The white bar at the bottom right equals 100 μm for the higher power photos on the right side. The same white bar equals 200 μm for the lower power left side photos. PMM: punctate midline myelotomy; OA: osteoarthritis; NeuN: neuronal nuclei; IBA1: ionized calcium-binding adaptor molecule 1; DAPI: 4′, 6-diamidino-2-phenylindole

Immunohistochemical staining for microglia with ionized calcium-binding adaptor molecule 1 (IBA1) [22] indicated the presence of microglia associated with the dorsal column lesion site (Figure 5, green), while there was no staining evident for astrocytes using an antibody against astrocyte-specific glial fibrillary acidic protein (GFAP) [23] two weeks post-dorsal column lesion (data not shown). The spinal cord neurons were identified by neuronal nuclei (NeuN) staining (Figure 5, red) and all nuclei were counterstained with DAPI (4′, 6-diamidino-2-phenylindole) (Figure 5, blue).

 

## Discussion

This study was intended to probe the question, "Does the postsynaptic dorsal column pathway (PSDC) play a role in conveying pain signals from the axial spine to more rostral levels of the nervous system?" If so, spine pain signals should be reduced by PMM. The behavioral testing indicates both secondary mechanical and heat hypersensitivity develop in both hind paws within three days after induction of the spine OA pain model with bilateral lumbar facet joint injections of uPA. The mechanical withdrawal thresholds and heat response latencies increased toward baseline after the PMM surgery and were significantly improved compared to unlesioned animals with OA. Without the PMM, this model of lumbar facet pain typically produces a hypersensitivity that persists at least four weeks [18-19]. The behavioral data and microglia accumulation at the PMM lesion site in the dorsal column provides an indication that the PSDC pathway is at least one of the routes signaling information about the back pain to higher brain centers.

The importance of understanding how spine pain signals ascend in the spinal cord relates to the importance of back, neck, and spine pain in the practice of medicine. Low back pain and cervical pain are leading causes of “years lived with disability” worldwide [16]. Knowing more about these pathways could lead to better treatments for chronic back, neck, and spine pain, especially at the “end-stage” of the severity spectrum when pain becomes disabling, intractable, and not well treated by any current means. Put in broad perspective, back pain is the second most common reason patients seek the help of a doctor, after “cold” and “flu” symptoms. Although most episodes of back pain are self-limited, and some are symptomatic of other illnesses requiring specific treatment, the vast majority of persistent chronic back pain seen by medical professionals (primary care physicians, chiropractors, neurosurgeons, and orthopedic spine surgeons) comes from degenerative changes in the discs and/or facet joints. To the extent that these degenerative changes lead to instability or to the nerve root, cauda equina, or spinal cord compression, surgery for decompression and/or fusion may be indicated and effective. That still leaves a large number of patients with primary degenerative “axial” spine pain for whom surgery is only questionably effective or was already tried with inadequate relief or even worsening [24-26]. Such patients often descend slowly into progressive opiate drug dependence, inability to work, and/or inactivity. As their pain-related inactivity and quality of life worsen further, obesity and depression are common [27]. An estimated 70% of all spine procedures are performed for axial spine pain alone, and approximately 48% of the money spent by employer health plans on opiate prescriptions was for low back pain [25]. The problem can be even worse for the estimated 50% of patients with back pain who report no improvement even when given potent opioids [26]. Many of the prescription pain medicines that reach the “street” and lead to opiate addiction were originally prescribed for chronic spine pain. In the United States, the incidence of low back pain is over 10% of the population, with lifetime incidence between 51% to 84% [27]. The costs to society of these chronically painful degenerative spine conditions are enormous, amounting to an estimated annual cost between $80 to $102 billion in 2005. Additionally tragic is that many patients become disabled by axial spine pain alone, without major numbness or weakness, and could otherwise be much more active and functional. Alternatives to chronic opiate therapy for end-stage degenerative spine conditions would be a welcome development with implications not only for alleviating human suffering but also for improving the negative societal consequences of chronic opiate therapy. As the population ages, this becomes increasingly true.

How might information gained from these studies be applied clinically?

A more detailed knowledge of the spinal cord pathways serving spine pain would improve our chance of finding an effective means to alleviate the severe intractable chronic pain commonly seen in the aging and/or injured degenerating spine and with cancer metastatic to the spine. If it can be shown that the PSDC pathway is a major contributor to the conduction of spine pain, then it may be possible to surgically interrupt or otherwise manipulate the pathway pharmacologically or by focused electrical stimulation in order to achieve pain relief. Currently, dorsal column stimulation (DCS) is used to treat some forms of spine pain, but the method appears to be most effective for radicular pain and is not typically effective for axial spine pain [28-29]. The exact structures stimulated with the relatively crude epidural electrodes typically used are not well understood and probably not very specific [29]. It is also incompletely known whether and which specific spinal cord pathways may be “turned on” or “off” by the stimulation. Knowing more about the specific pathways involved may suggest refinements in these stimulation methods that are both more selective and more effective for the types of axial spine pain that are so common and problematic.

Surgical interruption of the PSDC, as performed in PMM or by other midline myelotomy, is already recognized as an effective therapy for severe intractable visceral pain [5-10, 30]. Here, we show that spine pain follows the same trajectory through the spinal cord as visceral pain, and the remarkably effective, durable, and minimal side effects characteristic of PMM might safely be applied to the much more common problem of severe intractable axial spine pain. The safety and effectiveness of PMM derive from a fortunate combination of factors. First, the PSDC pathway to be interrupted in PMM is located in the most surgically accessible and easily identifiable part of the spinal cord, the dorsal midline. Second, there are no descending motor pathways in the dorsal columns. This advantage is very different from the anterolateral quadrant of the spinal cord where the STT is comingled with descending motor pathways related to, among others, respiratory drive and micturition control. Anterolateral cordotomy, as a result, was typically only performed unilaterally to treat contralateral extremity pain, severely limiting its clinical usefulness, and it is rarely performed today. Third, presynaptic tactile and proprioceptive sensory pathways in the dorsal columns are relatively tolerant of the type of limited lesion performed in PMM [7]. Fourth, preventing spine pain signals from reaching the brainstem and thalamus could reduce the descending facilitation contribution to chronic pain. 

## Conclusions

The purpose of this study was to clarify how pain signals originating from the spine ascend in the spinal cord to reach higher levels of the nervous system. The data presented here provide the first evidence that pain signals originating from the spine itself are transmitted, at least in part, in the postsynaptic dorsal column pathway, and that such pain signals are subject to interruption by punctate midline myelotomy. Further studies will be needed to clarify the extent to which the spine pain signals travel in the PSDC pathway in comparison to the classical STT anterolateral quadrant systems. This will be a crucial question to answer because if the PSDC is the dominant pathway for pain originating in the spine, then it may be possible to develop effective strategies for manipulating this pathway selectively for the control of intractable “end stage” spine pain.
